# The optimal antithrombotic strategy for post-stroke patients with atrial fibrillation and extracranial artery stenosis—a nationwide cohort study

**DOI:** 10.1186/s12916-024-03338-7

**Published:** 2024-03-13

**Authors:** Chuan-Tsai Tsai, Yi-Hsin Chan, Jo-Nan Liao, Tzeng-Ji Chen, Gregory Y. H. Lip, Shih-Ann Chen, Tze-Fan Chao

**Affiliations:** 1 Division of Cardiology, Department of Medicine, Taipei Veterans General Hospital, No. 201, Sec. 2, Shih-Pai Road, Taipei, Taiwan; 2 Institute of Clinical Medicine, and Cardiovascular Research Center, National Yang Ming Chiao Tung University, Taipei, Taiwan; 3 The Cardiovascular Department, Chang Gung Memorial Hospital, Linkou, Taoyuan Taiwan; 4 College of Medicine, Chang Gung University, Taoyuan, Taiwan; 5 Microscopy Core Laboratory, Chang Gung Memorial Hospital, Linkou, Taoyuan Taiwan; 6 Department of Family Medicine, Taipei Veterans General Hospital, Taipei, Taiwan; 7 Liverpool Centre for Cardiovascular Science, University of Liverpool & Liverpool Heart and Chest Hospital, Liverpool, UK; 8 Aalborg Thrombosis Research Unit, Department of Clinical Medicine, Aalborg University, Aalborg, Denmark; 9 Cardiovascular Center, Taichung Veterans General Hospital, Taichung, Taiwan

**Keywords:** Atrial fibrillation, Warfarin, NOAC, Stroke, Net clinical benefit

## Abstract

**Background:**

In post-stroke atrial fibrillation (AF) patients who have indications for both oral anticoagulant (OAC) and antiplatelet agent (AP), e.g., those with carotid artery stenosis, there is debate over the best antithrombotic strategy. We aimed to compare the risks of ischemic stroke, composite of ischemic stroke/major bleeding and composite of ischemic stroke/intracranial hemorrhage (ICH) between different antithrombotic strategies.

**Methods:**

This study included post-stroke AF patients with and without extracranial artery stenosis (ECAS) (*n* = 6390 and 28,093, respectively) identified from the Taiwan National Health Insurance Research Database. Risks of clinical outcomes and net clinical benefit (NCB) with different antithrombotic strategies were compared to AP alone.

**Results:**

The risk of recurrent ischemic stroke was higher for patients with ECAS than those without (12.72%/yr versus 10.60/yr; adjusted hazard ratio [aHR] 1.104, 95% confidence interval [CI] 1.052–1.158, *p* < 0.001). For patients with ECAS, when compared to AP only, non-vitamin K antagonist oral anticoagulant (NOAC) monotherapy was associated with lower risks for ischaemic stroke (aHR 0.551, 95% CI 0.454—0.669), the composite of ischaemic stroke/major bleeding (aHR 0.626, 95% CI 0.529—0.741) and the composite of ischaemic stroke/ICH (aHR 0.577, 95% CI 0.478—0.697), with non-significant difference for major bleeding and ICH. When compared to AP only, warfarin monotherapy was associated with higher risks of major bleeding (aHR 1.521, 95% CI 1.231—1.880), ICH (aHR 2.045, 95% CI 1.329—3.148), and the composite of ischaemic stroke and major bleeding. With combination of AP plus warfarin, there was an increase in ischaemic stroke, major bleeding, and the composite outcomes, when compared to AP only. NOAC monotherapy was the only approach associated with a positive NCB, while all other options (warfarin, combination of AP-OAC) were associated with negative NCB.

**Conclusions:**

For post-stroke AF patients with ECAS, NOAC monotherapy was associated with lower risks of adverse outcomes and a positive NCB. Combination of AP with NOAC or warfarin did not offer any benefit, but more bleeding especially with AP-warfarin combination therapy.

**Supplementary Information:**

The online version contains supplementary material available at 10.1186/s12916-024-03338-7.

## Background

Vascular disease (whether coronary, carotid, or peripheral artery disease) is commonly present in patients with atrial fibrillation (AF) and may be evident as prevalent or newly diagnosed in the post-stroke setting. Secondary prevention requires oral anticoagulant (OAC) to prevent recurrent stroke due to AF, but for vascular disease in the non-AF setting, anti-platelet agents (AP) are often prescribed to prevent recurrent stroke.

Thus, in stable post-stroke AF patients with indications for both OAC and antiplatelet treatment, e.g. those with associated vascular diseases, there is debate over the best antithrombotic strategy [1, 2]. There are some data for AF patients with stable coronary artery disease from observational cohorts and randomised trials [3, 4], but data for AF patients with carotid or vertebral artery disease are limited. Indeed, about one in 10 patients with AF have extracranial artery stenosis (ECAS), and vice versa, and non-stenotic carotid artery disease is present in about half of AF patients [5]. Such patients are commonly treated with AP alone, or if OAC is used, some physicians may consider the combination of AP and OAC. However, in the era whereby non-vitamin K antagonist oral anticoagulant (NOAC) is the preferred stroke prevention strategy for AF patients [2], there are limited data for the use of NOACs in post-stroke AF patients with ECAS.

In this nationwide cohort study, our aim was to examine outcomes in post-stroke AF patients with ECAS. Furthermore, we aimed to explore stroke and bleeding outcomes as well as the net clinical benefit (NCB) with OAC (NOAC or warfarin) and with AP-OAC combination therapy, when compared to AP alone.

## Methods

### Data source

This study used the “National Health Insurance Research Database (NHIRD)” provided by the Health and Welfare Data Science Centre (HWDC), Ministry of Health and Welfare (MOHW), Taiwan. The National Health Insurance (NHI) system is a mandatory universal health insurance program that offers comprehensive medical care coverage to all Taiwanese residents. NHIRD consists of detailed health care data from over 23 million enrollees, representing more than 99% of Taiwan’s population. In this cohort dataset, the patients’ original identification numbers have been encrypted to protect their privacy, but the encrypting procedure was consistent, so that a linkage of the claims belonging to the same patient was feasible within the NHI database and can be followed continuously. The descriptions about Taiwan NHIRD have been reported in our previous studies [6–13].

### Study population

**Fig. 1:**
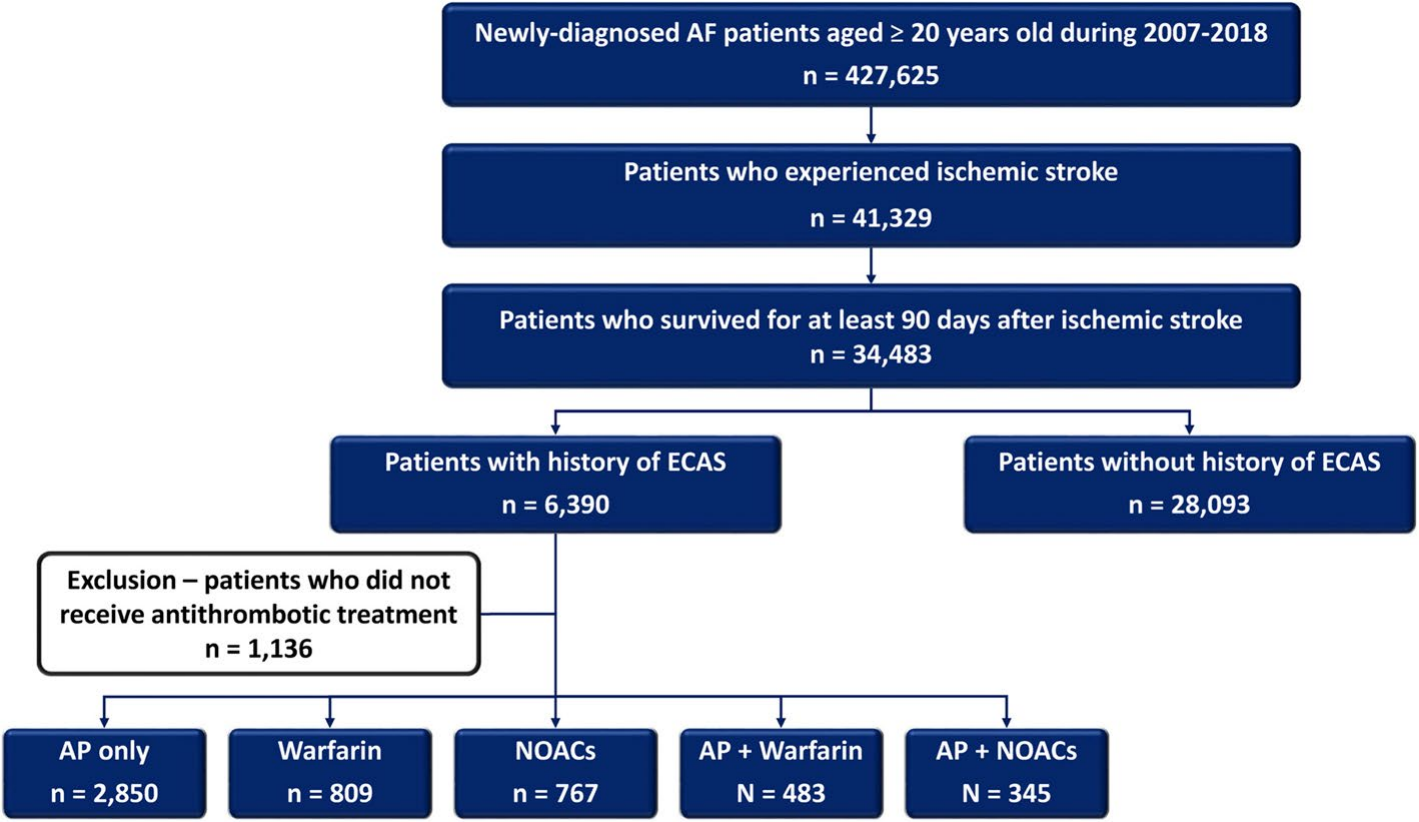
Study concept and the flowchart of the enrollment of study population. AF = atrial fibrillation; AP = anti-platelet agents; ECAS = extracranial artery stenosis NOACs = non–vitamin K antagonist oral anticoagulants

The flowchart of patient enrollment is shown in Fig. 1. From January 1st, 2007 to December 31st, 2018, a total of 427,625 newly-diagnosed AF patients aged ≥ 20 years were identified from the NHIRD. AF was diagnosed using the International Classification of Diseases (ICD), Ninth Revision, Clinical Modification (ICD-9-CM) codes (427.31) registered by the physicians responsible for the treatments of patients. The diagnostic accuracy of AF using this definition in NHIRD has been validated previously [14]. Among these patients, 34,483 of them who experienced ischemic stroke and survived for at least 90 days thereafter have constituted the study population. The index date was defined as the date when ischemic stroke occurred. The risk of further ischemic stroke was compared between patients with (*n* = 6,390) or without (*n* = 28,093) history of ECAS.

### Stroke prevention strategies after ischemic stroke among AF patients with history of ECAS

Among the 6,390 patients with history of ECAS, 1,136 of them who did not receive any antithrombotic treatments after ischemic stroke were excluded. The remaining 5,254 were categorized into 5 groups based on the stroke prevention strategies they received; that is, “AP” (*n* = 2,850), “warfarin” (*n* = 809), “NOAC” (*n* = 767), “AP plus warfarin” (*n* = 483) and “AP plus NOAC” (*n* = 345). The risks of clinical events of patients in different treatment groups were compared to those who received AP (reference group).

### Calculation of scores and definitions of clinical endpoints

The calculation rules of CHA_2_DS_2_-VASc score, HAS-BLED score and the definitions of clinical endpoints have been published in our previous works [15, 16]. Notably, the component of “labile international normalized ratio (INR)” was excluded from the calculation of HAS-BLED score in the present study because the information on INR of warfarin was not available in the Taiwan registry database. Also, abnormal renal and liver function were defined by the ICD-9-CM codes rather than laboratory data.

The clinical endpoints of the present study included the occurrences of ischemic stroke, major bleeding, intracranial hemorrhage (ICH), composite events of ischemic stroke or major bleeding, and ischemic stroke or ICH. The accuracy of diagnosis of ischemic stroke in Taiwan’s NHIRD has been reported to be around 94% [17]. Another validation study also demonstrated that the diagnostic accuracy of ischemic stroke in NHIRD was high, with the positive predictive value and sensitivity of 88.4% and 97.3%, respectively [18]. Major bleeding was defined as ICH or bleeding from gastrointestinal or genitourinary or respiratory tract requiring hospitalization [19]. Patients were followed up from the index date to the occurrence of mortality or December 31st, 2018, whichever occurred first.

### Falsification analysis

In order to further assess the likelihood of confounding by indication, we analyzed three falsification endpoints (cellulitis, colon cancer and extremity fracture/dislocation) which were unlikely to be affected by different stroke prevention strategies. A finding of an association between different stroke prevention strategies and these falsification endpoints would therefore indicate the presence of unmeasured confounders. On the contrary, if risks of these falsification endpoints of different patient groups did not differ significantly, the differences between different stroke prevention strategies with regard to clinical outcomes in which we were interested may be less likely due to treatment selection bias.

### Analysis of net clinical benefit

The NCB for different stroke prevention strategies compared with AP was calculated using the formula: (ischemic stroke rate on AP minus ischemic stroke rate on certain stroke prevention strategy) – weighting factor x (ICH rate on certain stroke prevention strategy minus ICH rate on AP). The weighting factor reflects the relative impact, in terms of death and disability, of experiencing an ICH versus experiencing an ischemic stroke [20–22]. The NCB with 95% confidence intervals (CI) were calculated from rate differences of ischemic stroke and ICH of the present study based on the weights previously produced and reported in the studies by Singer et al. [20], Connolly et al. [21], and Lip et al. [22]. A positive NCB favors certain stroke prevention strategy (i.e. NOACs), when compared to AP.

### Statistical analysis

Data are presented as the mean value and standard deviation (SD) for continuous variables, and proportions for categorical variables. Differences between continuous values and nominal variables were assessed using the unpaired two-tailed *t*-test and chi-squared test, respectively. The incidences of clinical events were calculated from dividing the number of events by person-time at risk. The risks of adverse events were assessed using the Cox regression analysis adjusted for age, sex and clinical variables which were significantly different among the groups. The proportional hazards assumption was tested using Schoenfeld residual test which showed no non-proportionality. All statistical significances were set at a *p* < 0.05.

## Results

The clinical characteristics of patients with or without ECAS are shown in Table 1. Patients with ECAS were slightly older (77.55 versus 76.75 years old, *p* < 0.001) and had more comorbidities, except for heart failure, compared to those without. Males were more prevalent in ECAS group. Overall, the CHA_2_DS_2_-VASc scores of 2 groups were similar (5.92 versus 5.91, *p* = 0.515). The risk of recurrent ischemic stroke was higher for patients with ECAS than those without (12.72%/yr versus 10.60/yr, adjusted hazard ratio [aHR] 1.104, 95% CI 1.052—1.158, *p* < 0.001) after a median follow-up duration of 3.49 years (interquartile range 1.52–5.60 years).

**Table 1:** Baseline characteristics of AF patients with or without history of ECAS

Variables	ECAS ( +) *n* = 6,390	ECAS (-) *n* = 28,093	*P* value
Age, years; mean value (SD)	77.55 (9.93)	76.75 (10.64)	< 0.001
Age ≥ 75 years, n (%)	4274 (66.89)	17966 (63.95)	< 0.001
Age 65–74 years, n (%)	1409 (22.05)	6272 (22.33)	0.632
Male gender, n (%)	3923 (61.39)	14451 (51.44)	< 0.001
Comorbidities, n (%)			
Hypertension	5798 (90.74)	24660 (87.78)	< 0.001
Diabetes mellitus	2743 (42.93)	11610 (41.33)	0.019
Congestive heart failure	3002 (46.98)	14333 (51.02)	< 0.001
Vascular diseases	1092 (17.09)	3372 (12)	< 0.001
COPD	2098 (32.83)	8084 (28.78)	< 0.001
Hyperlipidemia	3321 (51.97)	11355 (40.42)	< 0.001
Autoimmune diseases	355 (5.56)	1207 (4.3)	< 0.001
Cancer	832 (13.02)	3060 (10.89)	< 0.001
Hyperthyroidism	205 (3.21)	733 (2.61)	0.013
Abnormal renal function	1559 (24.4)	5401 (19.23)	< 0.001
Abnormal liver function	1065 (16.67)	4123 (14.68)	< 0.001
Anemia	1028 (16.09)	4351 (15.49)	0.233
History of bleeding	2104 (32.93)	8825 (31.41)	0.020
Alcohol excess/abuse	130 (2.03)	462 (1.64)	0.043
Gout	1648 (25.79)	6326 (22.52)	< 0.001
CHA_2_DS_2_-VASc score; mean values (SD)	5.92 (1.36)	5.91 (1.38)	0.515
HAS-BLED score, mean value (SD)	4.14 (1.13)	3.91 (1.11)	< 0.001

*AF* Atrial fibrillation, *COPD* chronic obstructive pulmonary disease, *ECAS* Extracranial artery stenosis, *SD* Standard deviation

### Antithrombotic strategies and clinical events in AF patients with ECAS

Table 2 shows the baseline characteristics of AF patients with ECAS receiving different antithrombotic therapies after ischemic stroke. Patients receiving AP only were older and had higher mean CHA_2_DS_2_-VASc and HAS-BLED scores compared to other groups.

**Table 2:** Baseline characteristics of patients with history of ECAS in different treatment groups

Variables	AP only *n* = 2,850	Warfarin* n = 809	NOACs^#^ *n* = 767	AP + Warfarin^&^ *n* = 483	AP + NOACs^$^ *n* = 345	P*	P^#^	P^&^	P^$^
Age, years; mean value (SD)	78.92 (9.36)	74.84 (10.43)	77.48 (10.18)	74.77 (10.22)	76.07 (9.08)	< 0.001	< 0.001	< 0.001	< 0.001
Age ≥ 75 years, n (%)	2075 (72.81)	468 (57.85)	503 (65.58)	273 (56.52)	200 (57.97)	< 0.001	< 0.001	< 0.001	< 0.001
Age 65–74 years, n (%)	549 (19.26)	203 (25.09)	165 (21.51)	124 (25.67)	113 (32.75)	< 0.001	0.175	0.003	< 0.001
Male gender, n (%)	1774 (62.25)	457 (56.49)	473 (61.67)	317 (65.63)	254 (73.62)	0.004	0.771	0.150	< 0.001
Comorbidities, n (%)									
Hypertension	2648 (92.91)	686 (84.8)	704 (91.79)	437 (90.48)	321 (93.04)	< 0.001	0.307	0.087	0.928
Diabetes mellitus	1283 (45.02)	270 (33.37)	327 (42.63)	209 (43.27)	172 (49.86)	< 0.001	0.237	0.475	0.091
Congestive heart failure	1320 (46.32)	401 (49.57)	352 (45.89)	249 (51.55)	140 (40.58)	0.103	0.835	0.034	0.041
Vascular diseases	546 (19.16)	86 (10.63)	119 (15.51)	92 (19.05)	84 (24.35)	< 0.001	0.015	0.955	0.033
COPD	1006 (35.3)	217 (26.82)	252 (32.86)	118 (24.43)	116 (33.62)	< 0.001	0.203	< 0.001	0.535
Hyperlipidemia	1497 (52.53)	369 (45.61)	474 (61.8)	253 (52.38)	250 (72.46)	< 0.001	< 0.001	0.953	< 0.001
Autoimmune diseases	166 (5.82)	28 (3.46)	46 (6)	23 (4.76)	31 (8.99)	0.002	0.858	0.319	0.049
Cancer	375 (13.16)	84 (10.38)	103 (13.43)	58 (12.01)	58 (16.81)	0.026	0.845	0.476	0.085
Hyperthyroidism	102 (3.58)	28 (3.46)	15 (1.96)	10 (2.07)	9 (2.61)	0.872	0.008	0.041	0.300
Abnormal renal function	785 (27.54)	129 (15.95)	167 (21.77)	114 (23.6)	99 (28.7)	< 0.001	< 0.001	0.062	0.655
Abnormal liver function	476 (16.7)	110 (13.6)	150 (19.56)	67 (13.87)	70 (20.29)	0.026	0.074	0.101	0.116
Anemia	505 (17.72)	102 (12.61)	97 (12.65)	51 (10.56)	33 (9.57)	< 0.001	< 0.001	< 0.001	< 0.001
History of bleeding	938 (32.91)	219 (27.07)	272 (35.46)	127 (26.29)	104 (30.14)	0.001	0.189	0.003	0.293
Alcohol excess/abuse	45 (1.58)	18 (2.22)	23 (3.0)	9 (1.86)	2 (0.58)	0.257	0.031	0.476	< 0.001
Gout	731 (25.65)	192 (23.73)	222 (28.94)	129 (26.71)	121 (35.07)	0.262	0.072	0.627	< 0.001
CHA_2_DS_2_-VASc score; mean values (SD)	6.06 (1.3)	5.63 (1.39)	5.86 (1.42)	5.77 (1.43)	5.83 (1.32)	< 0.001	< 0.001	< 0.001	0.002
HAS-BLED score, mean value (SD)	4.63 (0.95)	3.29 (1.04)	3.64 (1.01)	4.37 (0.98)	4.61 (0.92)	< 0.001	< 0.001	< 0.001	0.688

*AP* Anti-platelet agents, *COPD* Chronic obstructive pulmonary disease, *ECAS* Extracranial artery stenosis, *NOACs* Non–vitamin K antagonist oral anticoagulants, *SD* Standard deviation

^*^P value between Warfarin and AP only

^#^*P* value between NOACs and AP only

^&^*P* value between “AP + warfarin” and AP only

^$^*P* value between “AP + NOACs” and AP only

**Fig. 2:**
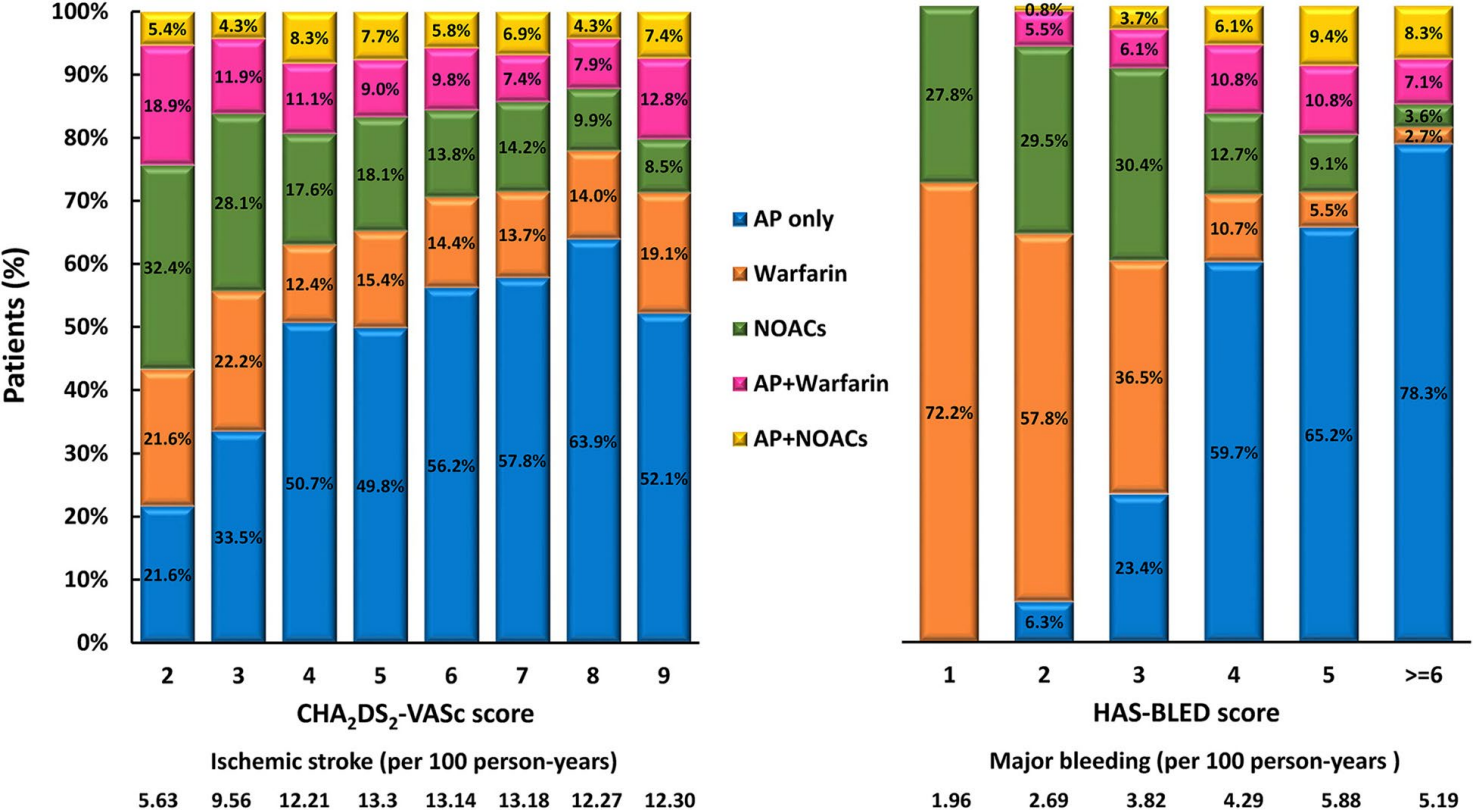
Distributions of different stroke prevention strategies in different CHA_2_DS_2_-VASc and HAS-BLED scores. AP = anti-platelet agents; NOACs = non–vitamin K antagonist oral anticoagulants

Figure 2 shows the event rates in different CHA_2_DS_2_-VASc and HAS-BLED scores. As expected, the risks of ischemic stroke and major bleeding were higher as the scores increased. Figure 2 also demonstrates the distributions of different stroke prevention strategies in relation to different risk score points. The percentages of OAC use decreased and the proportions of “AP only” increased in groups with a higher HAS-BLED score (Fig. 2). Differently, the percentages of OAC (warfarin or NOAC) use were discordantly higher in groups with lower CHA_2_DS_2_-VASc scores (Fig. 2).

**Fig. 3:**
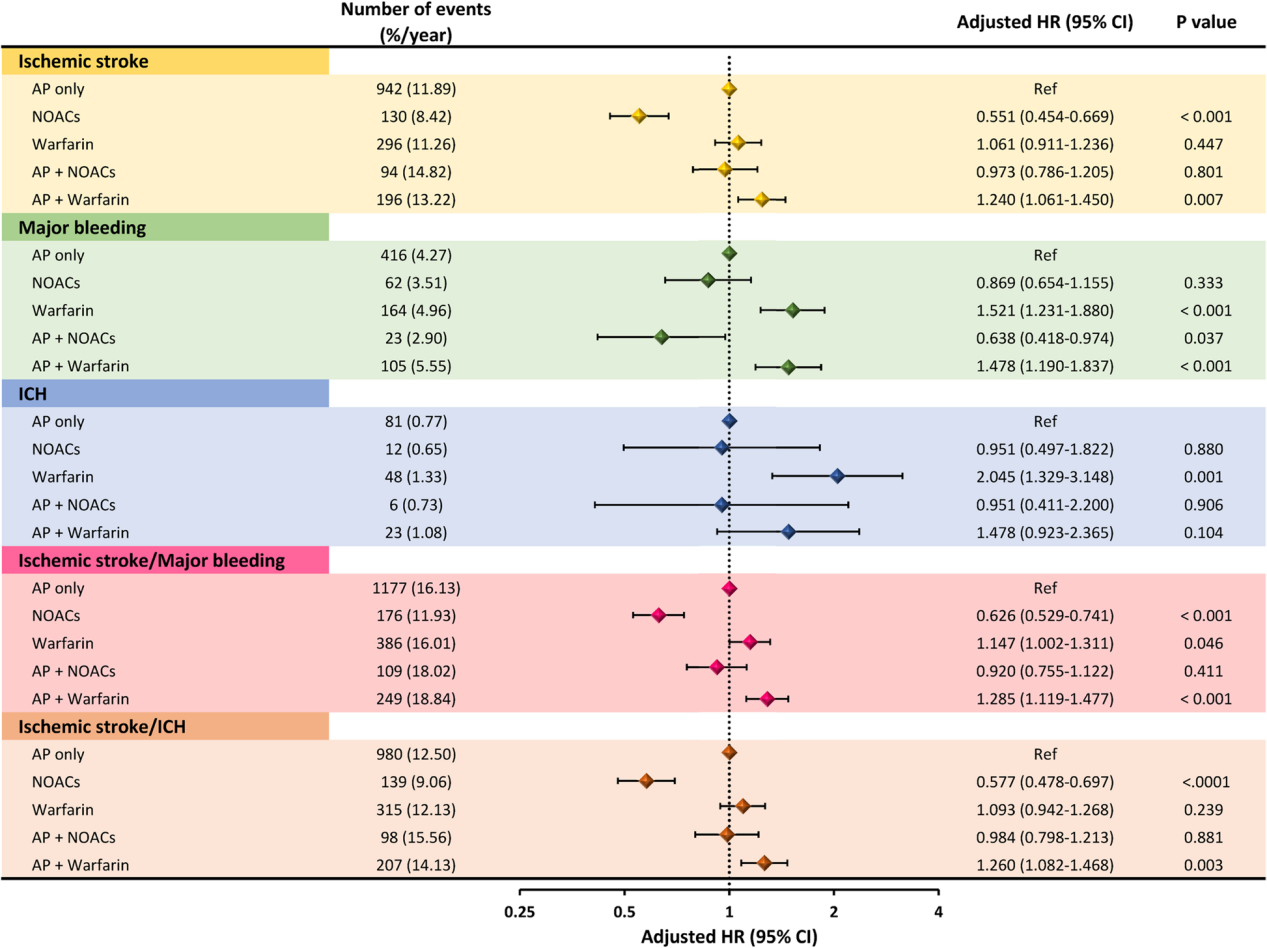
Risks of clinical events of patients receiving different stroke prevention strategies compared to “AP only”. AP = anti-platelet agents; HR = hazard ratio; ICH = intra-cranial hemorrhage; CI = confidence interval; NOACs = non–vitamin K antagonist oral anticoagulants

**Fig. 4:**
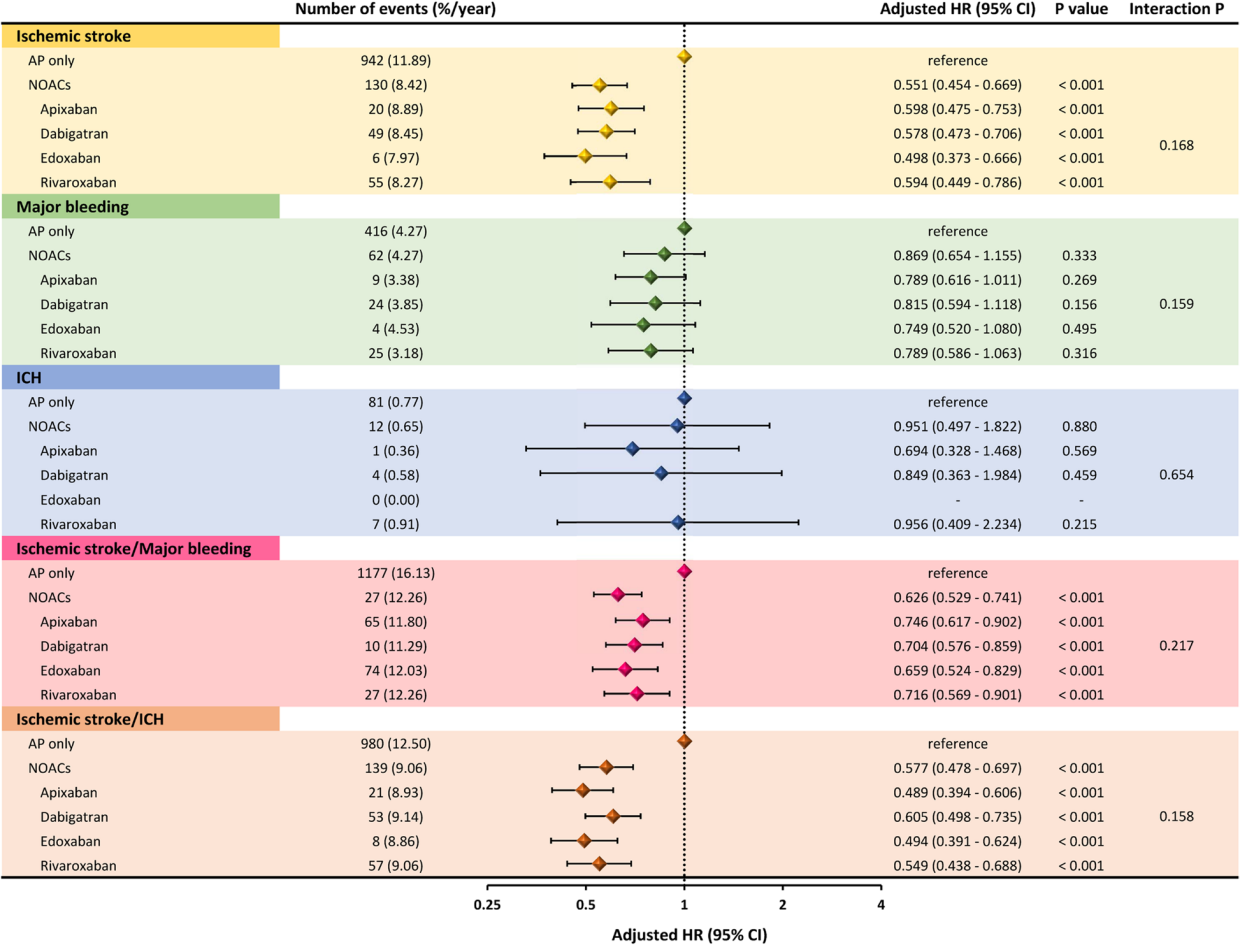
Risks of clinical events of patients receiving different NOACs compared to “AP only”. AP = anti-platelet agents; HR = hazard ratio; ICH = intra-cranial hemorrhage; CI = confidence interval; NOACs = non–vitamin K antagonist oral anticoagulants

When compared to AP only (reference), NOAC monotherapy was associated with lower risks for ischaemic stroke (aHR 0.551, 95%CI 0.454—0.669), the composite of ischaemic stroke/major bleeding (aHR 0.626, 95%CI 0.529—0.741) and the composite of ischaemic stroke/ICH (aHR 0.577, 95%CI 0.478—0.697), with nonsignificant difference for major bleeding and ICH (Fig. 3). With combination of AP plus NOAC, there was no difference in ischaemic stroke, ICH or the two composite outcomes when compared to AP only. The clinical outcomes of NOACs versus “AP only” were generally consistent for each of the different NOACs (interaction *P* values > 0.05 for each clinical events) (Fig. 4).

When compared to AP only, warfarin monotherapy was associated with higher risks of major bleeding (aHR 1.521, 95% CI 1.231—1.880), ICH (aHR 2.045, 95% CI 1.329—3.148), and the composite of ischaemic stroke and major bleeding (Fig. 3). The combination of AP plus warfarin was associated with higher risks of ischaemic stroke (aHR 1.240, 95% CI 1.061—1.450), major bleeding (aHR 1.478, 95% CI 1.190—1.837), the composite of ischaemic stroke/major bleeding (aHR 1.285, 95% CI 1.119—1.477) and the composite of ischaemic stroke/ICH (aHR 1.260, 95% CI 1.082—1.468) when compared to AP only (Fig. 3).

### Sensitivity analysis

The sensitivity analysis was performed to focus on patients who survived longer than 1 year after the index date and consider mortality as the competing risk in Cox regression models. The results were generally consistent to that of the principal analysis (Additional file 1: Figure S1).

### NCBs of different stroke prevention strategies versus “AP only”

Table 3 summarises the NCB of different stroke prevention strategies compared to AP only. NOAC monotherapy was the only approach associated with a positive NCB (irrespective of weighting definition), while all other options (warfarin, combination AP-OACs) were associated with negative NCB.

**Table 3:** Net clinical benefits for each treatment according to different weight models

Stroke prevention strategy	NCB based on different weight models, % per year (95% CI)		
	Relative weight of ICH compared to ischemic stroke according to Singer et al.[20] Weight = 1.5	Relative weight of ICH compared to ischemic stroke according to Connolly et al.[21] Weight = 3.08	Relative weight of ICH compared to ischemic stroke according to Lip et al.[22] Weight = 2.44
Compared to AP (Reference group)	–	–	–
NOACs	3.65 (3.32 to 3.98)	3.84 (3.63 to 4.05)	3.76 (3.45 to 4.07)
Warfarin	-2.13 (-2.28 to -1.98)	-2.46 (-2.56 to -2.36)	-2.32 (-2.45 to -2.19)
AP + NOACs	-2.87 (-3.00 to -2.74)	-2.81 (-2.90 to -2.72)	-2.83 (-2.94 to -2.72)
AP + Warfarin	-1.80 (-1.95 to -1.64)	-2.28 (-2.39 to -2.18)	-2.09 (-2.22 to -1.95)

*AP* Anti-platelet agents, *CI* Confidence interval, *ICH* Intra-cranial hemorrhage, *NCB* Net clinical benefit, *NOACs* Non–vitamin K antagonist oral anticoagulants

### Falsification analysis

The risks of 3 falsification endpoints did not differ significantly between different stroke prevention strategies compared to “AP only” (Additional file 2: Table S1). The results of falsification analyses suggested that the significant differences between different treatment groups with regard to clinical outcomes in which we were interested may be less likely due to treatment selection bias.

## Discussion

In this nationwide study, our principal findings are as follows: (i) the overall risk of recurrent ischemic stroke was higher for post-stroke AF patients with ECAS than those without; (ii) when compared to AP only, NOAC monotherapy in AF patients with ECAS was associated with lower risks for ischaemic stroke, the composite of ischaemic stroke/major bleeding and the composite of ischaemic stroke/ICH. With NOAC-AP combination therapy, there were no differences in ischaemic stroke, ICH or the two composite outcomes compared to AP only. Clinical outcomes were generally consistent for each different NOACs; (iii) when compared to AP only, warfarin monotherapy in AF patients with ECAS was associated with more major bleeding, ICH and the composite of ischaemic stroke/major bleeding. With combination of warfarin plus AP, there was an increase in ischaemic stroke, major bleeding, the composite of ischaemic stroke/major bleeding and the composite of ischaemic stroke/ICH, as well as a nonsignificant trend for more ICH; and (iv) NOAC monotherapy was the only approach associated with a positive NCB, while all other options (warfarin, combination of AP plus OACs) were associated with negative NCB.

As far as we are aware, this is the largest series of post-stroke AF patients with ECAS, where we clearly show the higher risk of recurrent ischemic stroke in post-stroke AF patients with ECAS than those without. Vascular disease is an independent predictor for ischaemic stroke in AF patients [23–25], although most prior studies have focused on coronary or peripheral artery disease of the lower limbs and not ECAS per se. Our study clearly highlights the need for a comprehensive evaluation of the post-stroke AF patient, to include an assessment of ECAS. In a systematic review and meta-analysis, the pooled prevalence of carotid artery stenosis in AF patients was 12.4% (95% CI 8.7–16.0%), with reported prevalence ranging from 4.4% to 24.3% [5].

In the presence of ECAS, many clinicians treat patient with AP alone, or if OAC is used, they would commonly add AP to OAC. For primary stroke prevention in AF patients with asymptomatic carotid artery disease, antiplatelet therapy is sometimes combined with OAC although evidence from large RCTs is lacking. Hence, our study provides important insights into the optimal antithrombotic therapy strategy for various clinical outcomes, in the secondary prevention setting of AF patients with ECAS. We found that NOACs monotherapy in post-stroke AF patients with ECAS was associated with lower risks for ischaemic stroke, the composite of ischaemic stroke/major bleeding and the composite of ischaemic stroke/ICH, when compared to AP only. However, when AP is added to NOAC (as the combination therapy), there was no advantage for ischaemic stroke, ICH or the two composite outcomes compared to AP only. The clinical outcomes were generally consistent for each different NOACs.

In contrast, warfarin monotherapy in AF patients with ECAS was associated with more major bleeding, ICH and the composite of ischaemic stroke/major bleeding, but no reduction in ischaemic stroke compared to AP only. With combination of warfarin plus AP, there was a large increase in ischaemic stroke, major bleeding, the composite of ischaemic stroke/major bleeding and the composite of ischaemic stroke/ICH, as well as a trend for more ICH. This would suggest that when OAC is considered, a NOAC would be a better option compared to warfarin. Whether NOAC or warfarin was used, outcomes were less good when the OAC was used in combination with AP therapy. Indeed, the NCB analysis was positive only for NOACs, and not for other antithrombotic therapy strategies for post-stroke AF patients with ECAS.

Our observations are consistent with data in patients with stable coronary artery disease. A meta-analysis by Lee et al. found no significant difference in major adverse cardiovascular events in patients with AF treated with OAC plus AP compared with those treated with OAC monotherapy (HR 1.09; 95%CI 0.92 to 1.29), but combination therapy was associated with a significantly higher risk of major bleeding, with no significant differences in rates of stroke and all-cause death [26]. In the AFIRE trial, NOAC monotherapy (with rivaroxaban) was noninferior to combination of NOAC plus AP for efficacy and superior for safety in patients with AF and stable coronary artery disease [4].

Our findings are also reinforced by NCB analyses with different weighting models, showing NOAC monotherapy was the only antithrombotic strategy associated with a positive NCB, while other approaches including warfarin monotherapy or OAC combinations with AP were associated with a negative NCB. Another implication from our data is to reinforce that efforts to mitigate bleeding risks are particularly important once combination therapy of AP-OAC was adopted. In a prospective cluster RCT of AF patients (with or without comorbid vascular disease), the strategy of corrections of modifiable bleeding risk factors and proactive follow-up for high bleeding risk patients resulted in less major bleeding at 1 year and an increase in OAC use [27].

Also, stroke prevention is only one aspect of the integrated approach to AF care, as reflected in the ABC (Atrial fibrillation Better Care) pathway which is now recommended in guidelines [2, 28]. The ABC pathway has been shown to reduce adverse outcomes in patients with AF in various studies [29–31]. Given the increasing focus on the post-stroke patient and their high cardiovascular risk [32], recent attention has also been directed towards a more holistic or integrated care approach to post-stroke management, which includes appropriate antithrombotic therapy, better functional and psychological status and cardiovascular risk factors/comorbidity optimization [33].

### Limitations

There are several limitations of the present study mainly owing to the nature of the database we used. First, the degree of ECAS was not recorded in our dataset, and therefore, whether the results of our study could be generalized to all patients with ECAS with different severities was unclear. Second, the diagnosis of AF and occurrence of ischemic stroke were based on the diagnostic codes registered by the physicians responsible for the treatments of patients; nonetheless, the accuracy of these diagnoses have been previously validated [14, 17, 18]. Third, information about the quality of anticoagulation control of warfarin, as reflected by the time in therapeutic range (TTR), was lacking in our dataset. In the RE-LY trial, the TTR for warfarin was only 44% in Taiwan [34], and whether well-managed warfarin could be associated with better clinical outcomes compared to AP in our study population is unclear. Also, data about the percentages of appropriate dosing of NOACs and the compliance/adherence of NOAC users were not available. The higher risk of ischemic stroke observed for the combination of “warfarin and AP” may be partly explained by an even lower international normalised ratio when clinical physicians adopted this strategy under the concern of bleeding. Furthermore, the higher risk of major bleeding with this combination may lead to the temporary discontinuation of all antithrombotic drugs once bleeding occurred which resulted in subsequent ischemic events. Fourth, since our study was an observational study rather than a randomized trial, the presence of unmeasured confounders and selection bias is highly probable which could confound the analyses. Although the results of falsification analyses may suggest that the significant differences between different treatment groups with regard to clinical outcomes in which we were interested may be less likely due to treatment selection bias, we can only report “associations” and do not imply causality. Fifth, our study was performed in an “intention to treat” design, and did not take the changes of stroke prevention strategies during the follow up into considerations. At the end of follow up, around 73.6% and 70.3% of patients initially categorized as “AP only” and “NOACs” groups were still under the same treatment, respectively. The persistence rate of NOACs was similar to that reported in prior real-word studies [35, 36] and ENGAGE AF-TIMI 48 trial [37]. Lastly, the present study only enrolled Taiwanese patients, and whether the results can be extrapolated to other populations remains uncertain. Owing to these limitations mentioned above, our findings should be regarded as “hypothesis generating” and would need to be confirmed in further large prospective randomised trials.

## Conclusions

Post-stroke AF patients with ECAS are at high risk of recurrent ischemic stroke. Compared to AP only, NOAC monotherapy was associated with lower risks of ischaemic stroke, the composite of ischaemic stroke/major bleeding and the composite of ischaemic stroke/ICH, with a positive NCB. In contrast, warfarin monotherapy was associated with more major bleeding, ICH and the composite of ischaemic stroke/major bleeding. Combination of AP with NOAC or warfarin did not offer any profound benefit, but more bleeding especially with AP-warfarin combination therapy.

### Supplementary Information

**Additional file 1:** **Fig. S1.** Risks of clinical events of patients receiving different stroke prevention strategies compared to “AP” only excluding patients experiencing mortality within 1 year and adjusting “mortality” as the competing risk.

**Additional file 2:** **Table S1.** Risks of 3 falsification endpoints of patients receiving different stroke prevention strategies compared to “AP only”.

## Data Availability

Please get in touch with the corresponding authors for more information.

## Abbreviations

ABC: Atrial fibrillation better care
AF: Atrial fibrillation
aHR: Adjusted hazard ratio
AP: Antiplatelet agent
CI: Confidence interval
ECAS: Extracranial artery stenosis
HWDC: Health and welfare data science centre
ICD: International classification of diseases
ICD-9-CM: International classification of diseases, ninth revision, clinical modification
ICH: Intracranial hemorrhage
INR: International normalized ratio
MOHW: Ministry of health and welfare
NCB: Net clinical benefit
NHI: National health insurance
NHIRD: National health insurance research database
NOAC: Non-vitamin K antagonist oral anticoagulant
OAC: Oral anticoagulant
SD: Standard deviation
TTR: Time in therapeutic range
