# Supplementary material for: The optimal antithrombotic strategy for post-stroke patients with atrial fibrillation and extracranial artery stenosis—a nationwide cohort study
Source: BMC Med. 2024 Mar 13;22:113. doi: 10.1186/s12916-024-03338-7 (PMC10935818; doi:10.1186/s12916-024-03338-7)

**Additional file 1: Fig S1. Risks of clinical events of patients receiving different stroke prevention strategies compared to “AP” only excluding patients experiencing mortality within 1 year and adjusting “mortality” as the competing risk.**


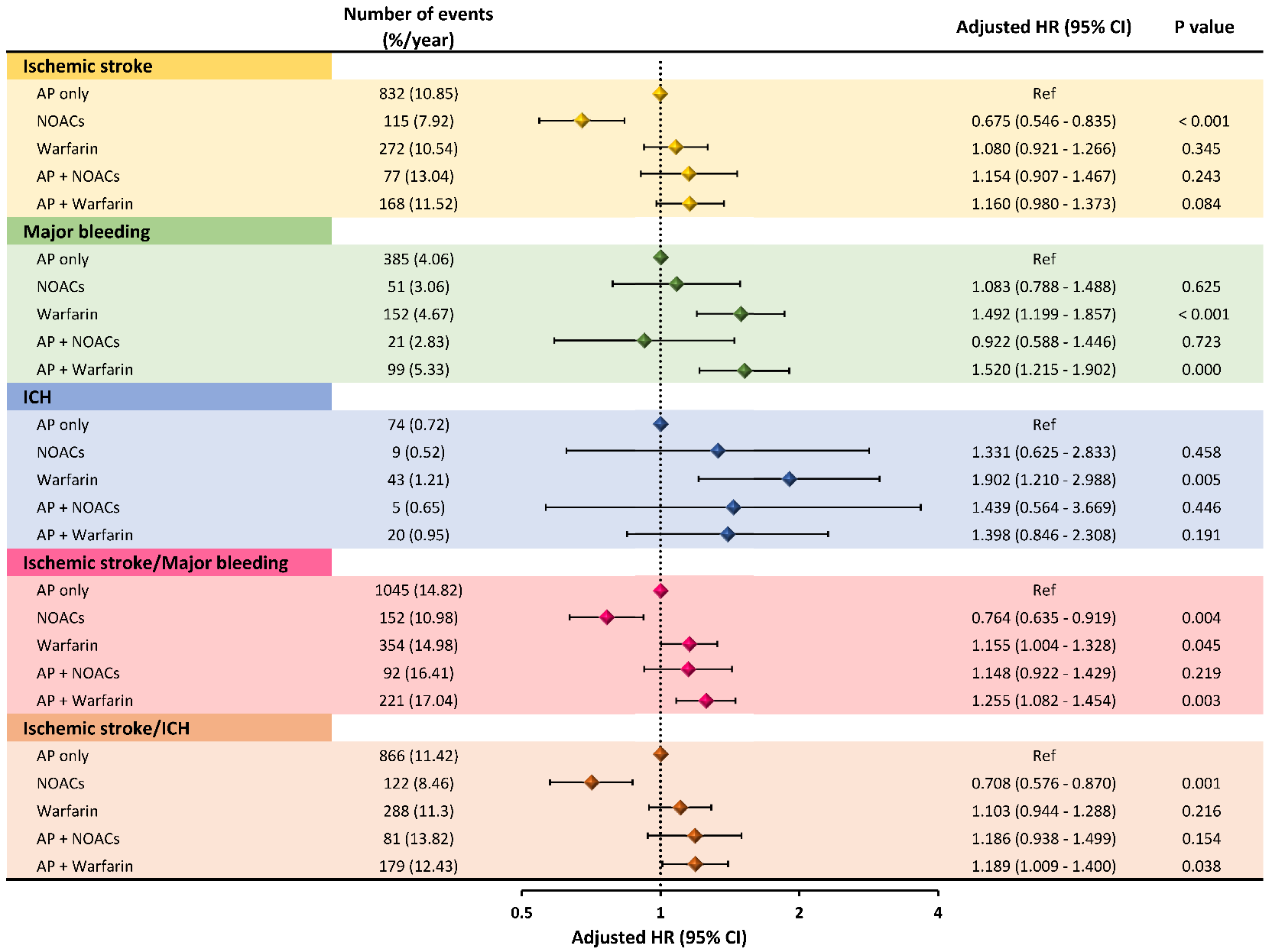

Supplement: Supplementary file 1 — Additional file 1: Fig. S1. Risks of clinical events of patients receiving different stroke prevention strategies compared to “AP” only excluding patients experiencing mortality within 1 year and adjusting “mortality” as the competing risk. [file 12916_2024_3338_MOESM1_ESM.docx]
