# Supplementary material for: The optimal antithrombotic strategy for post-stroke patients with atrial fibrillation and extracranial artery stenosis—a nationwide cohort study
Source: BMC Med. 2024 Mar 13;22:113. doi: 10.1186/s12916-024-03338-7 (PMC10935818; doi:10.1186/s12916-024-03338-7)
Supplement: Supplementary file 2 — Additional file 2: Table S1. Risks of 3 falsification endpoints of patients receiving different stroke prevention strategies compared to “AP only”. [file 12916_2024_3338_MOESM2_ESM.docx]

**Additional File 2: Table S1**

**Risks of 3 falsification endpoints of patients receiving different stroke prevention strategies compared to “AP only”**

|  | **Event rate**  **(%/year)** | **Adjusted HR (95%CI)** | **P value** |
| --- | --- | --- | --- |
| **Cellulitis** |  |  |  |
| AP only | 2.84 | Ref |  |
| NOACs | 2.45 | 0.978 (0.704 - 1.360) | 0.896 |
| Warfarin | 2.04 | 0.992 (0.749 - 1.312) | 0.954 |
| AP + Warfarin | 3.14 | 1.071 (0.710 - 1.616) | 0.743 |
| AP + NOACs | 2.17 | 0.829 (0.604 - 1.137) | 0.245 |
|  |  |  |  |
| **Colon cancer** |  |  |  |
| AP only | 0.20 | Ref |  |
| NOACs | 0.27 | 1.026 (0.721 - 1.458) | 0.873 |
| Warfarin | 0.33 | 1.135 (0.837 - 1.538) | 0.931 |
| AP + Warfarin | 0.36 | 1.035 (0.645 - 1.659) | 0.682 |
| AP + NOACs | 0.23 | 0.985 (0.692 - 1.401) | 0.209 |
|  |  |  |  |
| **Extremity fracture/dislocation** |  |  |  |
| AP only | 3.14 | Ref |  |
| NOACs | 3.15 | 0.986 (0.626 - 1.554) | 0.2943 |
| Warfarin | 2.57 | 0.864 (0.577 - 1.294) | 0.508 |
| AP + Warfarin | 3.21 | 1.055 (0.598 - 1.859) | 0.829 |
| AP + NOACs | 3.16 | 1.023 (0.572 - 1.828) | 0.270 |

AP = anti-platelet agents; CI = confidence interval; HR = hazard ratio; NOACs = non–vitamin K antagonist oral anticoagulants
